# Limb-girdle Muscular Dystrophy with New Mutation in Sarcoglycan Beta Gene: A Case Report

**Published:** 2018-12

**Authors:** Eskandar TAGHIZADEH, Hamed ABDOLKARIMI, Reza BOOSTANI, Arianeh SADRNABAVI

**Affiliations:** 1. Cellular and Molecular Research Center, Yasuj University of Medical Sciences, Yasuj, Iran; 2. Dept. of Medical Genetics, School of Medicine, Mashhad University of Medical Sciences, Mashhad, Iran; 3. Dept. of Biology, Science and Research Branch, Islamic Azad University, Tehran, Iran; 4. Dept. of Neurology, Faculty of Medicine, Mashhad University of Medical Sciences, Mashhad, Iran; 5. Dept. of Medical Genetics, Academic Centers for Education, Culture, and Research (ACECR), Mashhad, Iran; 6. Medical Genetic Research Center (MGRC), School of Medicine, Mashhad University of Medical Sciences, Mashhad, Iran

**Keywords:** Limb-girdle muscular dystrophy, Next-generation sequencing, Sarcoglycan beta gene

## Abstract

Limb-girdle muscular dystrophies (LGMDs) are a large group of genetic diseases in which there is muscle weakness and they are heterogonous diseases. The following study conducted in September 2017 in Mashhad, northwest of southern Khorasan Province, Iran reports a four years girl of autosomal recessive LGMD with proximal weakness and myopathy patterns. We detected four new alternations in this patient not reported for our population. One of them was important clinically that exists as unreported homozygous deletion encompassing exon 2 of the Sarcoglycan Beta (*SGCB*) gene. The use of Next Generation Sequencing (NGS) in the diagnosis of rare genetic pathologies is becoming ever more widespread in clinical practice. We used the NGS method for the first time to analysis the mutation in this family.

## Introduction

Limb-girdle muscular dystrophies (LGMDs) are a large group of genetic diseases in which there is muscle weakness and wasting and include more than 30 different inherited disorder mostly caused by genetics (1, 2). LGMDs can have different inherited pattern (3). Most forms of LGMDs are autosomal recessive and several rare forms are autosomal dominant (4). These disorders have various forms that they are caused by mutation in many different genes (5). In Iran is not possible to identify subtypes of the disease and the number of such patients is high. “The use of Next Generation Sequencing (NGS) in the diagnosis of rare genetic pathologies is becoming ever more widespread in clinical practice (6,7)”. We report the clinical features and mutational analysis of an Iranian patient with LGMD diseases.

## Case presentation

The study, conducted in 2017, describes a four-yr-old girl, third in birth rank from Ferdos (a city near Mashhad in the northwest of southern Khorasan Province), whose parents are first cousins. Her older brother suffers from congenital hearing loss. The parents first realized that the kid has below-average head circumference following a routine check-up at age one. Then, they were referred to a pediatric neurologist, who established microcephalus. After further investigation, she also has significant delay in achieving motor milestones, along with mild lexical difficulties. She was not diagnosed with a specific neurological disorder by age three. At present, aged 4, she was checked for metabolic disorders when the muscle and liver enzyme levels were high. Complete blood count, erythrocyte sedimentation rate (ESR), thyroid functions, lactate, pyruvate, ammonia, plasma, and amino acid profile were normal. Creatinine phosphokinase (CPK) was 1154 IU/l (Reference: 24–190 IU/l). Other abnormal test includes Aldolase 52 U/L (normal <14.5 U/L), total lactate dehydrogenase (LDH) 840 U/L (normal 143–290 U/L), aspartate aminotransferase (AST) 102 U/L (normal 8– 50 U/L), and alanine aminotransferase (ALT) 201 U/L (normal 7–45 U/L). She was subsequently referred for neuromuscular examination as well as EMG study. She is actually suffering from generalized hypotonia and weakness. Mental functions including speech and cranial nerves were normal in neurological examination. No skeletal abnormalities were observed. Moreover, EDX studies showed a Myopathic process with mild muscle irritability. Sensory system was normal and Respiratory, cardiovascular and abdominal systems were normal.

**Fig. 1:**
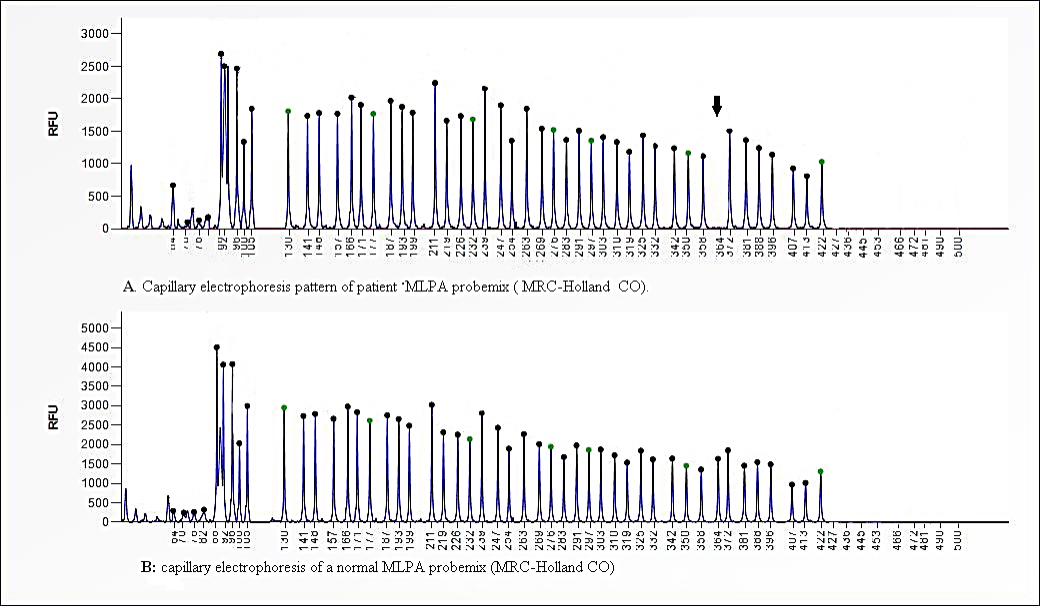
Detected mutations. A: Patient sample, B: Normal sample

Based on her proximal muscle weakness and other signs and symptoms we doubt to LGMD and offer molecular testing for those genes that involves in LGMDs. After explaining the NGSmethod a consent form was taken from the patient and her family. Then blood sampling was done and genomic DNA was extracted. The genomic DNA was sent to Germany country for Whole Exome Sequencing (WES). A panel of genes related to LGMD pathogenesis consists of The Anoctamin 5(*ANO5),* Calpain 3(*CAPN3),* Caveolin 3(*CAV3),* Dystroglycan 1(*DAG1),* DnaJ heat shock protein family (Hsp40) member B6(*DANJB6),* Dysferlin *(DYSF),* Fukutin related protein *(FKRP),* Fukutin *(FKTN),* Lamin A/C *(LMNA),* Myotilin *(MYOT),* Plectin *(PLEC),* Protein O-linked mannose N-acetylglucosaminyltransferase 1(*POMGNT1),* Protein O-mannosyltransferase 2(*POMT2),* Sarcoglycan Alpha *(SGCA),* Sarcoglycan Beta *(SGCB)* and Protein O-mannosyltransferase1(*POMT1) genes* were analyzed by PCR and Next Generation Sequencing of both DNA strands of the entire coding region and the highly conserved exon-intron splice junctions. NGS was done after Enrichment target areas by NimbleGen Sequence Capture Array (Roche, Madison). The results of NGS were analyzed based on reference genomes and databases including 1,000 genomes, Human Gene mutation Database (HGMD) and NCBI dbSNP. Pathogenicity prediction of these varieties was carried out by sifting mutation taster and Align-GVGD devices. Then new alternations were confirmed in her parents by Sanger sequencing. Moreover, MLPA kit used for large genomic deletions or duplications was performed to confirm the deletion of exon 2 the Sarcoglycan Beta (*SGCB) gene* (MRC-Holland MLPA, Lot 0708) (Fig. 1).

Ethics approval was not required but the consent form was received of patient and her parents.

## Results

The aim of NGS for this patient was to detection of most point mutations and small insertions or deletions (indels) known to occur in LGMD. For this purpose, NGS was done in a panel of genes that can have a role in LGMD pathogenesis. Targeted Exome Sequencing of both DNA strands of the entire coding region and the highly conserved exon-intron splice junctions in 16 genes listed in above shows 4 significant alternations in genome. These alternations are given below. After interpretation the new alternation in parent homozygous deletion encompassing exon 2 of the *SGCB* gene confirmed as main significant alternation in patients genome:

1- We detected a previously unreported heterozygous variant in exon 16 of the *POMT1* gene (c.1577G>A p.Arg526Gln). This heterozygous variant is present in her father but isn’t in her mothers
2- In addition, we detected unreported homozygous deletion encompassing exon 2 of the *SGCB* gene. Her parents are career for this deletion in exon 2.

We also detected the following to likely neutral variants:

3- A heterozygous variant in exon 5 of the *CANP3* gene (c.706G>A p.Ala236Thr)
4- A heterozygous mutation in intron 30 of the *PLEC* gene (c.4456-4A>G).

## Discussion

Limb-girdle muscular dystrophy may be transmitted in an autosomal recessive manner or less commonly in an autosomal dominant manner (5). Many genes can play a role in LGMD pathogenesis. Difficulties in accurate diagnosis and determination of inheritance in an individual family make genetic counseling particularly complicated (8, 9). LGMD has many genes the NGS can be the best choice for definitive diagnosis of LGMD genes.

We found four alternations includes a previously unreported heterozygous variant in exon 16 of the *POMT1* gene (c.1577G>A p.Arg526Gln), homozygous deletion encompassing exon 2 of the *SGCB* gene and two likely neutral variants includes a heterozygous variant in exon 5 of the *CANP3* gene (c.706G>A p.Ala236Thr) and a heterozygous mutation in intron 30 of the *PLEC* gene (c.4456-4A>G) but homozygous deletion in exon 2 of the *SGCB* gene is an important alternation that causes the diseases. *SGCB* gene is a subunit of a group of proteins called the sarcoglycan protein complex located in the membrane surrounding muscle cells. Mutation in *SGCB* gene especially exon deletions may prevent sarcoglycan complex from forming or from binding to the dystrophin complex and these changes reduce the strength and resilience of muscle fibers and thus can cause the LGMD phenotype (10). To date, only two large deletions have been reported in the HGMD professional database for *SGCB* gene. Mutations in this gene are associated with limb-girdle muscular dystrophy type 2E of inherited in an autosomal recessive manner. This deletion is present in parents with a heterozygous position and we conclude inheritance pattern is autosomal recessive. Parent of our case are carrier and they have a 25% chance for an affected child, a 50% chance of being an asymptomatic carrier, and a 25% chance of being unaffected and not a carrier. After interpretation these new alternations in patient and her parent homozygous deletion encompassing exon 2 of the *SGCB* gene confirmed as main significant alternation in patients genome. Furthermore, we can perform carrier testing and detect at-risk members for this family. We can perform prenatal diagnosis (PND) and Preimplantation genetic diagnosis (PGD) for couples that they are carrier. For her family, PND was done and a healthy child was born. Other found new alternations in this case discuses in below. We also detected heterozygous variant in exon 16 of the *POMT1* gene (c.1577G>A p.Arg526Gln) is located in a weakly conserved nucleotide and amino acid position. Analyzing this variant by sift mutation taster and Align-GVGD predicts this variant is probably benign. It is reported without any information on frequency in NCBI dbSNP. Mutations in *POMT1* gene are associated with muscular dystrophy-dystroglycanopathy type1A, 1B, 1C that all are autosomal recessive. This alternation is present in a heterozygous position and probably is not main reason our case diseases because it present in her father that is healthy but more research are needed in this area.

Heterozygous variant in exon 5 of the *CANP3* gene (p.Ala236Thr) is present in this case. This variant has previously been described diseases causing for limb-girdle muscular dystrophy. This variant is classified as polymorphism and HGMD professional 2013.3 has classified this variant from diseases causing mutation to likely disease-causing mutation and mentions that it possibly only deleterious in cis with Thr184Met. NCBI dbSNP reports it with a frequency of 0.2702 and the Exome Sequencing project describes it with a frequency of 0.0497 in the European American population and 0.3788 in African American population. This variant is a new variant in our population and we do not know its frequency.

Our analyzing for this case showed heterozygous mutation in intron 30 of the *PLEC* gene (c.4456-4A>G).NCBI dbSNP reports it with a frequency of 0.2310 and the Exome Sequencing Project describes it with a frequency of 0.2975 in the European American population and 0.0655 in the African American population and its frequency in our population is not determined because it is a newly found alternation. And we are requiring more study but we can detect this variant in members that they are at risk.

## Conclusion

Molecular biology has opened new method to understand the LGMD clinical diagnosis, classification, pathogenesis and treatment possibilities. However, our understanding of the pathophysiology of the majority of LGMD forms is in infancy but NGS is a new method for helping us.

## Ethical considerations

Ethical issues (Including plagiarism, informed consent, misconduct, data fabrication and/or falsification, double publication and/or submission, redundancy, etc.) have been completely observed by the authors.
